# From Diagnosis Delay to Targeted Therapy: A Retrospective Study of Pediatric DLGNT with a Comprehensive Literature Review

**DOI:** 10.3390/cancers18040549

**Published:** 2026-02-08

**Authors:** Riccardo De Carli, Viviana Minichini, Laetitia Lebrun, An Van Damme, Christophe Chantrain, Anais Fohn, Sandra Jacobs, Frederik De Smet, Pierre Leblond, Nicolas André, Pierluigi Calò

**Affiliations:** 1 Department of Pediatric Haematology and Oncology, Queen Fabiola Children Hospital, University Hospital of Brussels, 1020 Brussels, Belgium; 2 Service d’Hématologie et Oncologie Pédiatrique, University Hospital Timone, Assistance Publique-Hôpitaux de Marseille, 13385 Marseille, France; 3 Pediatric Hematology, Robert Debré Hospital, Assistance Publique-Hôpitaux de Paris, 75019 Paris, France; 4 Department of Neurosurgery, Erasme Hospital, University Hospital of Brussels, 1070 Brussels, Belgium; 5 Department of Pathology, Erasme Hospital, University Hospital of Brussels, 1070 Brussels, Belgium; 6 Department of Pediatric Haematology and Oncology, Cliniques Universitaires Saint-Luc, Université Catholique de Louvain, 1200 Brussels, Belgium; 7 Division of Pediatric Hematology-Oncology, Centre Hospitalier Chrétien (CHC), MontLégia, 4000 Liege, Belgium; 8 Department of Pediatric, Citadelle Hospital, 4000 Liege, Belgium; 9 Pediatric Hematology and Oncology, Department of Pediatrics, University Hospitals Leuven, 3000 Leuven, Belgium; 10 Laboratory for Precision Cancer Medicine, Translational Cell and Tissue Research Unit, Department of Imaging and Pathology, The Leuven Institute for Single Cell Omics (LISCO) and The Leuven Cancer Institute (LKI), KU Leuven, 3000 Leuven, Belgium; 11 Institut d’Hématologie et Oncologie Pédiatrique (iHOPE), Centre Leon Berard, 69008 Lyon, France; 12 Inserm CRO2 UMR_S 911, Aix-Marseille University, 13003 Marseille, France; 13 Metronomics Global Health Initiative, 13385 Marseille, France

**Keywords:** diffuse leptomeningeal glioneuronal tumors, DLGNT, low-grade glioma, *KIAA1549::BRAF* fusion, *MAPK* pathway, tovorafenib

## Abstract

**Background:** Diffuse leptomeningeal glioneuronal tumors (DLGNT) are rare pediatric central nervous system tumors, first recognized in the 2016 WHO classification. Their clinical course is highly heterogeneous, and no international consensus treatment guidelines are currently available. This study aims to describe clinical characteristics, disease evolution, and management strategies for pediatric DLGNT patients, with a focus on aggressive forms. **Methods:** This retrospective, multicenter, international study (Belgium and France) included pediatric patients diagnosed with DLGNT between 1 February 2016 and 31 December 2024. Clinical, radiological, histopathological, molecular, and therapeutic data were collected. Findings were analyzed and contextualized through an extensive literature review. **Results:** Eleven patients were enrolled (median age: 8.2 years; median follow-up: 52 months). The median delay between the first MRI and definitive diagnosis was 6.5 months. Symptoms of intracranially elevated pressure were present in 55% of patients. Two-thirds of the patients presented with leptomeningeal dissemination at diagnosis. The primary tumor site could not be identified in two patients. A *KIAA1549::BRAF* transcript fusion was detected in 82% of cases, and chromosome *1q* gain in 38%. All patients underwent surgery at diagnosis. The median number of therapeutic lines was four: 82% received chemotherapy (weekly vinblastine in 55%, vincristine/carboplatin regimen in 45%), 64% received *MAPK* pathway-targeted therapy, and 18% underwent radiotherapy. Five-year overall survival (OS) was 68.5%, and median progression-free survival (PFS) was 5.3 months after first-line therapy and 16.5 months after the second line. At the end of follow-up, only one patient achieved complete remission, and 78% of survivors presented with persistent neurological deficits. **Conclusions:** This study underscores the significant diagnostic delay, clinical heterogeneity, and absence of standardized therapeutic approaches in pediatric DLGNT patients. Conventional low-grade glioma chemotherapy constitutes the current treatment backbone, while *MAPK* pathway-targeted therapies show promising potential. Further studies and the establishment of an international registry are crucial to better characterize aggressive subtypes and optimize management strategies.

## Simple Summary

Diffuse leptomeningeal glioneuronal tumors are predominantly pediatric neoplasms that were recently incorporated into the WHO classification. Given their rarity and their variable clinical presentation and disease course, diagnosis is challenging, and standardized management guidelines are lacking. Conventional low-grade glioma chemotherapy remains the backbone of treatment; however, the identification of *MAPK* pathway activation in DLGNT has paved the way for the use of *MAPK*-targeted therapies. The aim of this study was to retrospectively describe a patient cohort, disease evolution, treatments, and outcomes. Our study provides a comprehensive overview of DLGNT and highlights diagnostic delay, clinical heterogeneity, the promising activity of tovorafenib in progressive disease, and the need for further studies to better characterize aggressive subtypes to optimize therapeutic strategies.

## 1. Introduction

The term diffuse leptomeningeal glioneuronal tumor was first proposed in 2010 for four pediatric cases with mixed neuronal and glial immunophenotypes, diffuse leptomeningeal dissemination without parenchymal mass, and an indolent clinical course, not corresponding to any entity in the World Health Organization (WHO) classification at that time [1]. Similar disorders, resembling oligodendrogliomas with leptomeningeal spread, have previously been described [2,3,4,5,6,7,8,9,10]. Since 2016, DLGNT have been formally recognized in the WHO classification of central nervous system (CNS) tumors [11]. Their incidence is unknown, but glioneuronal tumors overall represent around 3% of pediatric brain tumors [12]. The median age at diagnosis is four years, with a male predominance (ratio 1.7:1) [13,14,15,16]. To date, more than 170 pediatric and 37 adult cases have been reported [17,18]. There is no established correlation with cancer predisposition syndromes, although some cases associated with constitutional rasopathy disorders due to RAF1 germline mutation and with mismatch repair gene mutations (*MSH6*, *MLH1*, *MSH2*, *PMS2*) have been reported [19,20].

### 1.1. Clinical Findings

The clinical presentation is heterogeneous and non-specific. Signs of intracranial hypertension (ICH), such as headache and vomiting, are most common, followed by cranial nerve palsies, diplopia, ataxia, gait disturbance, back pain, limb weakness or pain, migraine, and hemiparesis. Seizures or altered consciousness may occur [1,12,14,21,22,23,24]. Disease progression is usually slow, although rapidly progressive forms leading to death within 24 months have been reported [14,20,25,26].

### 1.2. Radiological Findings

Radiologically, DLGNT display characteristic features on magnetic resonance imaging (MRI); T1 pre- and post-gadolinium, T2, and FLAIR sequences guide diagnosis and follow-up. The most common finding is diffuse leptomeningeal enhancement, particularly in the posterior fossa, brainstem, and along the spinal cord [24]. Nodular intracranial or intraspinal leptomeningeal thickening occurs in nearly two-thirds of patients, mainly at presentation, whereas cystic lesions tend to appear later [1,14]. T2-hyperintense nodular or cystic lesions along the subpial surface of the brain and spinal cord are typical of multifocal forms and are critical for differential diagnosis [13,24,27,28,29]. Leptomeningeal dissemination may be radiologically occult, as microscopic leptomeningeal involvement can exist despite a normal MRI [22,24,30]. Intra-parenchymal foci may also be observed, especially in the cervical spinal cord, cerebellum, brainstem, and hypothalamus [13,17]. These may represent primary or secondary lesions, or deep extensions from superficial leptomeningeal involvement [21]. Hydrocephalus occurs in 70–80% of patients at diagnosis and is believed to result from leptomeningeal thickening, which disrupts the blood–brain barrier and impairs cerebrospinal fluid (CSF) flow [17,22,31]. Initial differential diagnosis includes diffuse glioma, pilocytic astrocytoma, ganglioglioma, extraventricular neurocytoma, and leptomeningeal carcinomatosis, as well as non-malignant conditions such as bacterial or tuberculous meningitis, sarcoidosis, neurocysticercosis, and neurocutaneous syndromes [13,29,32,33,34].

### 1.3. Cytology, Histology, Molecular Biology and Methylation Profile

CSF analysis is rarely informative, with the only consistent abnormality being marked proteinorachia [1,5,21,31,35,36,37]. The absence of neoplastic cells can be explained by their entrapment within a dense leptomeningeal stroma, preventing their release into cerebrospinal fluid circulation [1,21]. Nevertheless, CSF evaluation remains useful in the initial workup to exclude other diagnoses [38].

Biopsy is essential but technically challenging, as specimens are often insufficient or non-representative [19,38]. Therefore, it should target an enhancing leptomeningeal area or, alternatively, a solid lesion that is easily accessible [39].

Histologically, nearly 70% of DLGNT display a desmoplastic stroma, occasionally myxoid or mixed, with low to moderate cellularity [21]. The monomorphic, low-grade tumor cells show oval nuclei with fine chromatin, a clear perinuclear halo, inconspicuous nucleoli, and a cytoplasm reminiscent of oligodendroglial cells [1,21,30,40]. Mitotic activity is generally low, although anaplastic features have been reported [1,21,24,25,41,42]. Immunohistochemically, OLIG2, MAP2, and S100 are strongly expressed, whilst GFAP and synaptophysin show variable expression [24,30]. NeuN, EMA, and *IDH1 R132H* are negatives, as are *IDH1/IDH2* mutations by molecular analysis [4,24,30,43]. Cytogenetically, the most common finding is the loss of chromosome *1p*, sometimes accompanied by *19q* codeletion [44,45]. Other abnormalities include *1q* gain, loss of chromosome 4, or loss of heterozygosity at *7q34-36* [24,46,47].

At the molecular level, aberrant activation of *MAPK/ERK* signaling predominates, especially via *KIAA1549::BRAF* fusion (60–80% of cases), while *BRAF V600E* mutation occurs in fewer than 5% of patients [19,24,45,46,48,49,50]. Furthermore, genome-wide DNA methylome profiling followed by a gene set enrichment analysis (GSEA) on 24 DLGNT samples revealed hyperactivation of multiple pathways beyond *MAPK*, including those involved in apoptosis, cell cycle regulation, T cell receptor (TCR) signaling, *MYC*, *WNT* and *p53* pathways [51]. Other anomalies include *NTRK2/3*, *RAF1* or *FGFR1* fusions [24,52]. *CDKN2A/B* deletions are exceptional, whereas *PDGFA/B* alterations have not been reported [24,53,54,55,56,57,58]. Liquid biopsy approaches are currently under investigation [59].

### 1.4. Management of DLGNT Patients

Currently, there is no standard treatment for DLGNT patients. European guidelines (ESCP) for the treatment of pediatric low-grade glioma (pLGG) and high-grade glioma (HGG) do not specifically address this entity [60,61]. Surgery is the cornerstone of initial management, although complete resection is rarely feasible owing to the leptomeningeal and metastatic spread present at diagnosis [17,51,62,63]. In minimally symptomatic and indolent cases, a watch-and-wait strategy is often considered [2,38]. Chemotherapy is generally the first-line treatment after surgery, particularly in younger children, to avoid or defer radiotherapy. Approximately 85% of patients receive chemotherapy, most commonly following pLGG protocols such as SIOP LGG 2004 (Vincristine/carboplatin combination) or weekly vinblastine (VBL) [17,64,65,66]. Other regimens are also proposed, such as temozolomide (TMZ), or combinations of etoposide, cyclophosphamide, and vincristine [19,21,36,38,40,49,67]. Since therapeutic response is variable, multiple lines of treatment are often required. Radiotherapy, mainly craniospinal (CS), is typically employed in cases of progressive or recurrent disease and appears to be more effective when administered earlier [21,49]. Between 2000 and 2021, more than one in five patients received CS irradiation [17].

Targeted therapies are playing an emerging role. In pLGG with *BRAF* mutation, the combination of dabrafenib and trametinib has demonstrated superior efficacy and tolerability compared with standard chemotherapy [68]. Following the encouraging results of the FIREFLY-1 phase 2 study, the ongoing LOGGIC/FIREFLY-2 phase 3 trial is evaluating tovorafenib versus standard chemotherapy as first-line treatment in pediatric LGG with *RAF* activation [69,70]. In DLGNT harboring *KIAA1549::BRAF* fusion or *BRAF V600E* mutation, *MEK* and *RAF* inhibitors have achieved prolonged disease control with moderate toxicity [70,71]. In relapsed diseases, agents such as bevacizumab, irinotecan, or everolimus have been used, with variable results [12,19].

### 1.5. Prognostic Factors

DLGNT are generally indolent tumors, with 5-year overall survival (OS) of 80% and 10-year OS of 70% [17,21]. Age at diagnosis above nine years, initial hydrocephalus, spinal localizations, and secondary dissemination have been associated with a poorer prognosis [17,27,49,65,72]. Histologically, the presence of mitoses, a *MIB-1* proliferation index ≥ 4%, and glomeruloid vascularization has also been linked to unfavorable outcomes [21].

In 2018, two molecular subgroups were identified through DNA methylation profiling: MC-1 and MC-2 [24]. Loss of chromosome *1p* is a consistent feature in both subgroups, whereas *1q* gain is present in all MC-2 cases and approximately one-third of MC-1 cases [24,73]. Clinically, MC-1 occurs in younger children (median age 5 years) and follows an indolent course, with 5-year overall survival (OS) and progression-free survival (PFS) rates of 100% and 86%, respectively. In contrast, MC-2 affects older patients (median age 14 years) and is associated with a poorer prognosis (5-year OS 43% and PFS 14%) [24,44,74].

The prognostic role of *1q* gain remains debated: while some studies indicate a 20-fold increased risk of progression, more recent research suggests that there is no correlation between *1q* status and patient outcome [51,60]. Regarding treatment, clinical evolution appears more favorable in patients receiving chemotherapy, while radiotherapy and surgery have no significant prognostic impact [17,40].

### 1.6. Objective of the Study

The primary objective of this retrospective study is to describe the demographic and clinical characteristics of a cohort of patients with DLGNT, as well as to analyze the management strategies employed. The collected data are compared with those reported in the literature, with a particular focus on aggressive forms to help identify the most effective therapeutic approaches.

## 2. Materials and Methods

This is a retrospective, multicentric, international study focusing on pediatric DLGNT patients (0–18 years). It includes patients diagnosed between 1 February 2016 and 31 December 2024 in a pediatric hematology and oncology center affiliated with the Belgian Society of Paediatric Haematology Oncology (BSPHO) or the French Society of Childhood Cancer (Société Française des Cancers de l’Enfant, SFCE). Histological diagnosis was required for inclusion, as well as a minimum follow-up period of six months. Patients with cancer predisposition syndrome were excluded. Medical records were reviewed to collect demographic, clinical, radiological, histological, molecular, and therapeutic data.

Primary tumor localization was defined by the presence of an intraparenchymal lesion. In patients with multiple lesions or isolated leptomeningeal infiltration without a parenchymal focus, the primary site was classified as “unidentified.” A lesion was considered metastatic if no anatomical contiguity with another lesion was present; in such cases, the dominant, measurable lesion was designated as the primary one. Treatment regimens and clinical complications were documented. Radiological responses were assessed according to Response Assessment in Pediatric Neuro-Oncology (RAPNO) criteria, when applicable [75,76]. In cases of predominant leptomeningeal disease, progression was defined by the appearance of a new area of enhancement, response by a reduction in the extent of enhancement, and stable disease by the absence of significant changes in contrast uptake, as determined by the reference neuroradiologist at each center. Surgery was considered a first-line treatment when it was not limited to biopsy alone or when biopsy was followed by a watch-and-wait strategy. Descriptive statistics were used to summarize patient and tumor characteristics. Overall survival (OS) was defined as the interval, expressed in years, between the date of diagnosis and death. Progression-free survival (PFS) was calculated for each line of treatment, from its initiation until disease progression or death. Patients alive at the time of analysis were censored at the date of last follow-up. Survival analyses were performed using the Kaplan–Meier method, and median survival times were reported with 95% confidence intervals.

Results were compared to the literature through a narrative review. A bibliographic search was conducted on PubMed and EMBASE, focusing on publications released between 2004 and 2024, and using the keywords “DLGNT” and “Diffuse Leptomeningeal Glioneuronal Tumor.”

## 3. Results

**Figure 1:**
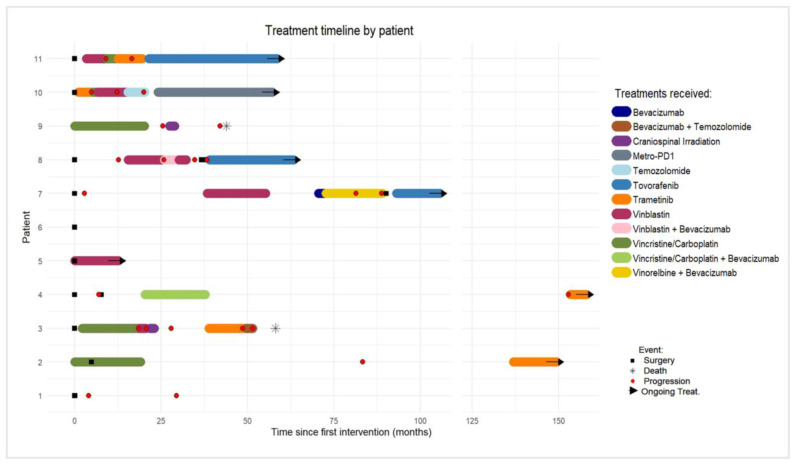
Treatment timeline by patient (swimmer plot).

**Figure 2:**
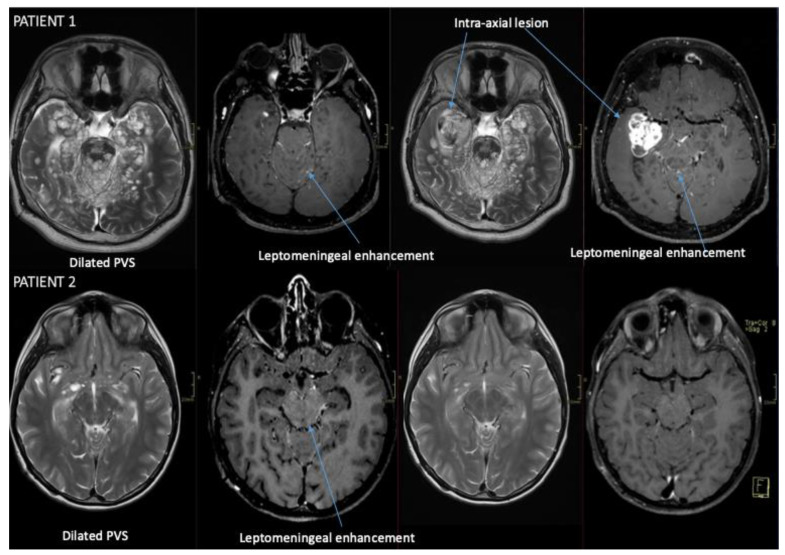
Radiological presentation and evolution of two patients with typical DLGNT findings. T2 turbo spin-echo (TSE) and T1 post-contrast black-blood sequences. *Patient 1*: Left panels: March 2023; right panels: July 2025. *Patient 2* Left panels: April 2021; Right panels: January 2022. *PVS*: perivascular spaces.

Eleven patients were included. Results are summarized in Table 1 and Figure 1; Figure 2 illustrates the radiological presentation and evolution of two patients. Males accounted for 36% of the cohort. Median age at diagnosis was 8.2 years (2.8–17.6) and 14.7 years at last follow-up (6.4–22.8), with a median follow-up of 52 months (6.0–146.0). The median interval between the first MRI and diagnosis was 6.5 months (0–120.0). ICP was present in 55% of patients at diagnosis, followed by ataxia (27%), lumbosacral pain (27%), and progressive neurological deficits (27%). One patient presented with meningism, nystagmus, torticollis, seizures, and behavioral abnormalities (9%). One patient had developmental delay and hemiparesis related to perinatal hypoxia. Thirty-six percent of the patients presented with an isolated spinal tumor. All MRIs were injected at diagnosis. T2-hyperintense nodular or cystic lesions were reported in four (36%) patients. In two patients (18%), it was not possible to locate the initial tumor site. Metastases were present in 72% of patients at diagnosis. One patient developed extracranial omental metastases. Leptomeningeal enhancement on MRI was observed in eight patients (72%), including one case in which dissemination appeared three years after diagnosis.

A molecular analysis was performed in ten patients (91%). *MAPK* pathway alteration was identified in nine patients (82%), exclusively through a *KIAA1549::BRAF* fusion, with no *BRAFV600E* mutations detected. Among the eight patients who underwent *1p*/*1q* analysis, six showed a *1p* deletion (75%) and three a *1q* gain (38%). DNA methylation profiling was available for only two patients: one classified as MC-1 and the other as MC-2.

The median number of therapeutic lines was four (1–7). Two patients (18%) had a macroscopically complete resection, four (36%) had a subtotal resection (STR), and four (36%) had biopsy alone. One patient (9%) underwent lobectomy for refractory epilepsy. A ventriculoperitoneal (VP) shunt was placed in four patients (36%) at diagnosis and in one patient (9%) during follow-up; two cases of device failure were documented. Four patients (36%) required at least two surgical procedures.

Nine patients (82%) received chemotherapy, seven of them as first-line adjuvant therapy. Four patients (36%) received at least three lines of chemotherapy. The most frequently used regimens were *VBL* (55%) and *SIOP LGG 2004* (45%). Two patients (18%) discontinued treatment due to toxicity, which included one case of reversible posterior encephalopathy syndrome (PRES) related to carboplatin and one case of unspecified vincristine toxicity.

Seven patients (64%) with *KIAA1549::BRAF* fusion transcript received *MAPK*-targeted therapy at different treatment lines: five received trametinib (45%) and three tovorafenib (27%), including one who received both. Bevacizumab was administered to five patients (45%), alone or in combination with other agents. One patient (9%) was enrolled in the *MetroPD1* trial combining the anti-PD1 nivolumab and metronomic chemotherapy, and two patients (18%) received craniospinal radiotherapy [74].

Median PFS was 5.3 months after the first treatment line (2–25), 16.5 months after the second line (3–83), 2.9 months after the third line (1–132), and 5.9 months after the fourth treatment line (4–16). Median PFS after *VBL* was 5.7 months (1–43) and 21 months (16–83) after *SIOP LGG 2004*. Median PFS was 7.1 months (4–11) after trametinib, whereas PFS was 100% after tovorafenib with a median follow-up of 19 months (7–25). Median PFS was 10.4 months (6.2–14.6) after CS irradiation. Three-year OS was 100%, and five-year OS was 68.5%. Two patients died (18%), 45 and 56 months after diagnosis. One patient (9%) was in complete remission (CR), three patients (27%) in partial response (PR), four patients (36%) in stable disease (SD), and one patient (9%) in progressive disease (PD) at the last follow-up. Among survivors, seven out of nine (78%) had sequelae at the last follow-up: chronic headache (27%), ataxia (18%), paresthesias (18%), emotional blunting (9%), intellectual disability (18%), epilepsy (9%), and chronic back pain (9%).

One patient with a *1q* gain died without achieving a therapeutic response, while the other two are in PR with vinblastine and tovorafenib, respectively.

## 4. Discussion

Identified as a distinct entity in 2016, DLGNT mainly affects children and adolescents, with a growing number of reported cases in recent years [26,45,77]. Their characterization continues to evolve, and they exemplify an integrated diagnostic approach that combines histological assessment with molecular and epigenetic analyses, with methylation profiling occupying a central role [52].

Deletion of *1p* is the most frequent cytogenetic alteration, observed in 75% of our patients [44]. In our cohort, *1q* gain was present in 38% of patients, whereas its prevalence is estimated at approximately 50% in published series, and it has been proposed as a key factor influencing disease evolution [46,49,74]. Deng et al. identified two methylation-based subgroups of DLGNT: MC-1 (*1q* gain in 35% of cases) and MC-2 (*1q* gain in 100%), with MC-1 showing significantly better OS and EFS [24]. Conversely, Chiang et al., comparing MC-1 and MC-2 with and without *1q* gain, demonstrated that OS and PFS were significantly reduced in the presence of *1q* gain, irrespective of methylation class [46]. In contrast, in the cohort reported by Mikkelsen et al., *1q* gain did not show any prognostic impact [51]. Altogether, the prognostic role of *1q* gain remains uncertain and requires further clarification. Since it might help identify patients at high risk of progression, its systematic assessment could be valuable, although it is not currently included among the diagnostic criteria [53].

*MAPK* pathway analysis is essential for establishing a diagnosis and for delineating therapeutic targets. In our cohort, 82% of patients harbored a *KIAA1549::BRAF* fusion, consistent with previously published series [46,49]. This alteration arises from the replacement of the N-terminal portion of *BRAF* by *KIAA1549*, leading to constitutive activation of the protein [78,79]. Its presence in both the glial and neuronal components suggests that it may represent an early oncogenic event acting as a common driver of tumor development [47]. No *BRAFV600E* mutations were detected, confirming their rarity in DLGNT [21,49].

Clinical manifestations are heterogeneous, and the long interval between the first neuroimaging and the final diagnosis—a median of 6.5 months in our cohort—highlights the clinical and radiological complexity of DLGNT [1,19,21].

Four patients showed no leptomeningeal enhancement at diagnosis, including one who developed dissemination three years later. This suggests that DLGNT without leptomeningeal involvement may be more common than previously estimated [13,30]. Consequently, the absence of leptomeningeal dissemination does not rule out the diagnosis, which should therefore be systematically considered in the differential diagnosis of localized CNS tumors.

DLGNT may present as spinal, supratentorial, infratentorial, or mixed disease, with the spinal cord representing the most frequent intraparenchymal site [19,21,36,38]. Determining the primary lesion can be challenging, particularly when multiple parenchymal foci are present at diagnosis. If lesions differ in consistency, the solid component is more likely to represent the primary focus, while a cystic lesion may correspond to secondary involvement [21]. MRI interpretation becomes even more complex when leptomeningeal disease is present. Several published cases describe the emergence of a parenchymal mass following an initially isolated leptomeningeal presentation, possibly reflecting tumor propagation through Virchow–Robin spaces from an originally leptomeningeal process [1,2].

In our cohort, a primary localization was identified in 82% of patients. Extra-CNS dissemination is exceedingly rare, yet one patient presented with histologically confirmed omental metastasis, representing the only case reported to date [58]. Other metastatic sites that have been described—such as lungs and bone marrow—were not identified in our series [80].

Evaluating tumor progression is problematic due to the lack of standardized DLGNT-specific radiological criteria. RAPNO criteria, developed for HGG, are useful when assessing an isolated lesion or, in the case of multifocal disease, when a predominant lesion is present. By contrast, they are not suitable for evaluating diffuse leptomeningeal involvement [76]. In this context, centralized review of imaging appears particularly relevant. Nevertheless, comparisons between patients remain difficult owing to the heterogeneity of therapeutic approaches and the timing of different treatment lines.

In the absence of international guidelines, the optimal therapeutic approach has not been defined. To date, only chemotherapy seems to be associated with a significant prognostic benefit [17,40,51]. All our patients underwent surgery as first-line management, either for diagnostic confirmation or for therapeutic purposes. Surgery maintains a central role, though complete resection is exceptional [81]. One patient was treated exclusively with lobectomy for refractory epilepsy, and in another, given the indolent evolution and accessibility of the mass, sequential partial debulking procedures were favored over chemotherapy. Thus, medical treatment may be deferred or avoided in selected cases [2,38].

Chemotherapy was administered to 82% of our patients, consistent with other series [17]. The most frequently used regimens were *SIOP LGG 2004* and *VBL*, the standard adjuvant therapies in pLGG [65,66]. Despite the limited sample size, our findings suggest greater efficacy of *SIOP LGG 2004*—with a median PFS of 21 months—compared with 5.7 months for vinblastine. Superior activity of carboplatin-based chemotherapy has also been reported by other authors [82]. Bevacizumab was commonly employed as second-line therapy, either alone or in combination with chemotherapy (50% of cases).

The demonstration of *MAPK* pathway activation has supported the use of *MEK*, *RAF*, or pan-*RAF* inhibitors, with encouraging responses, mostly as disease stabilization [71,83]. In our cohort, seven patients (64%) received *MAPK*-targeted therapy: trametinib (n = 5) and tovorafenib (n = 3), with one patient receiving both agents after progression under trametinib. Trametinib, with a median PFS of 7.1 months, did not appear more effective than chemotherapy. Conversely, all patients treated with tovorafenib obtained sustained disease control, with a 100% PFS at a median follow-up of 19 months. These observations suggest that tovorafenib might represent a promising therapeutic option in DLGNT. However, long-term outcome data remain limited. Depending on the results of the LOGGIC/FIREFLY 2 trial for pLGG, earlier introduction of tovorafenib in *RAF*-activated DLGNT could be justified [70]. No association has been established between *BRAF* activation and prognosis, although the presence of an actionable target may broaden therapeutic options. Moreover, evidence that multiple signaling pathways are concurrently activated in DLGNT suggests that multi-pathway inhibition may be required [51]. One patient was treated within the metro-PD1 phase I study (NCT03585465) as a sixth-line therapy, with no disease progression or adverse effects after 18 months of follow-up. Although it is the only reported case to date, metronomic chemotherapy in association with nivolumab could be considered for refractory DLGNT, given its safety profile [84].

Craniospinal radiotherapy is reserved for refractory disease [17,21]. Two patients (18%) were given radiation therapy. Its toxicity—particularly on neurocognitive development—limits its use in younger children, although it remains a reasonable option in adolescents. Despite the radiosensitivity of pLGG, radiotherapy does not appear to improve OS in DLGNT, possibly because it is typically administered late in progressive forms, whereas earlier use might enable better disease control [17].

DLGNT have poorer long-term outcomes than other pLGG, whose 10-year OS is approximately 85% and even higher for pilocytic astrocytoma [17,85]. In our cohort, the 3-year OS was 100%, decreasing to 68.5% at 5 years from diagnosis. Nonetheless, progression occurred after nearly every treatment line, regardless of the regimen, except in the three patients receiving tovorafenib. At the last follow-up, only one patient was in CR (after lobectomy), seven were in SD or PR, and one was in PD, while two patients had died.

Patients receive multiple lines of therapy, with a median of four in our cohort, resulting in cumulative toxicity and substantial impact on quality of life. This highlights the need to prioritize treatments with low long-term toxicity. Among the nine survivors, seven (78%) presented neurological sequelae.

Our study has several limitations, primarily due to its retrospective design and the small sample size, which preclude robust statistical analyses and limit the generalizability of the findings. Importantly, further data are needed to assess the potential efficacy of tovorafenib in DLGNT. Notably, methylation status was available for only two patients, preventing comparison between MC-1 and MC-2, especially in terms of outcomes and response to MAPK-targeted therapy.

## 5. Conclusions

DLGNT remain poorly characterized. The high rate of progression and the difficulty in achieving complete remission reflect the absence of effective therapies. The establishment of an international registry integrating clinical and biological data would facilitate identification of risk factors and optimization of management strategies: standard-risk patients could benefit from pLGG-based protocols, whereas high-risk patients might require treatment intensification.

## Figures and Tables

**Table 1:** Clinical, radiological, cytogenetic and molecular features of the patients.

Patient	Sex	Age at Diagnosis (Y)	FU Duration (Mo)	Symptoms at Diagnosis	MRI-Diagnosis Delay (Mo)	Tumor Localization	LM Enhancement	MAPK Alteration	Del 1p	Gain 1q	Type of Surgery	VP Shunt	N of TRT Lines	Last FU Status
1	M	14.2	35	Headache, lombosacral pain	0	S + ST	Yes	*KIAA1549::BRAF*	Yes	No	B	Yes	2	PR
2	F	3.1	146	Headache, ataxia	120	S + ST + IT	No	*KIAA1549::BRAF*	No	No	B	Yes	3	SD
3	M	11.9	56	Headache, vomiting, limb pain	0	S + ST	No	*KIAA1549::BRAF*	No	No	B	No	6	D
4	M	5.9	133	Headache, vomiting, ataxia, nystagmus, seizures, progressive neurological deficit	24	S + ST + IT	Yes	*KIAA1549::BRAF*	Yes	No	STR	Yes	4	PD
5	F	11.3	6	Lombosacral pain	15	S	Yes	*KIAA1549::BRAF*	Yes	Yes	MCR	No	2	PR
6	F	12.0	29	Seizures		ST	Yes	*U*	U	U	MCR	No	1	CR
7	M	6.4	100	Ataxia, progressive neurological deficit, lombosacral pain	84	S	Yes	*KIAA1549::BRAF*	U	U	MCR	No	6	SD
8	F	17.6	62	Progressive neurological deficit	0	S + IT	Yes	*KIAA1549::BRAF*	U	U	STR	Yes	7	PR
9	F	8.2	45	Headache	9	S	No	*No*	Yes	Yes	B	Yes	2	D
10	F	4.5	52	Headache	0	S + ST + IT	Yes	*KIAA1549::BRAF*	Yes	No	STR	No	6	SD
11	F	2.8	43	Torticolis	4	S	Yes	*KIAA1549::BRAF*	Yes	Yes	STR	No	5	SD

B: biopsy; CR: complete response; D: deceased; F: female; FU: Follow-up; IT: infratentorial; LM: Leptomeningeal; M: male; Mo: Months; MCR: macroscopically complete resection; PD: progressive disease; PR: partial response; S: spinal; ST: supratentorial; STR: subtotal resection; SD: stable disease; TRT: treatments; U: unknown; Y: years.

## Data Availability

The original contributions presented in this study are included in the article. Further inquiries can be directed to the corresponding author.
